# Mapping Glaucoma Patients' 30-2 and 10-2 Visual Fields Reveals Clusters of Test Points Damaged in the 10-2 Grid That Are Not Sampled in the Sparse 30-2 Grid

**DOI:** 10.1371/journal.pone.0098525

**Published:** 2014-06-20

**Authors:** Ryo Asaoka

**Affiliations:** Department of Ophthalmology, The University of Tokyo Graduate School of medicine, Tokyo, Japan; Massachusetts Eye & Ear Infirmary, Harvard Medical School, United States of America

## Abstract

**Purpose:**

To cluster test points in glaucoma patients' 30-2 and 10-2 visual field (VF) (Humphrey Field Analyzer: HFA, Carl Zeiss Meditec, Dublin, CA) in order to map the different regions damaged by the disease.

**Method:**

This retrospective study included 128 eyes from 128 patients. 142 total deviation (TD) values (74 from the 30-2 VF and 68 from the 10-2 VF) were clustered using the ‘Hierarchical Ordered Partitioning And Collapsing Hybrid – Partitioning Around Medoids’ algorithm. The stability of the identified clusters was evaluated using bootstrapping.

**Results:**

65 sectors were identified in total: 38 sectors were located outside the 10-2 VF whereas 29 sectors were located inside the 10-2 VF (two sectors overlap in both grids). The mapping of many sectors appeared to follow the distribution of retinal nerve fiber bundles. The results of bootstrapping suggested clusters were stable whether they were outside or inside the 10-2 VF.

**Conclusion:**

A considerable number of sectors were identified in the 10-2 VF area, despite the fact that clustering was carried out on all points in both the 30-2 VF and 10-2 VF simultaneously. These findings suggest that glaucomatous central VF deterioration cannot be picked up by the 30-2 test grid alone, because of poor spatial sampling; denser estimation of the central ten degrees, than offered by the 30-2 test grid alone, is needed. It may be beneficial to develop a new VF test grid that combines test points from 30-2 and 10-2 VFs – the results of this study could help to devise this test grid.

## Introduction

Glaucoma is one of the leading causes of blindness in the world [1], [2]. Glaucomatous visual field (VF) damage usually initiates in the mid-peripheral VF while the central region tends to be preserved until late on in the disease process. In advanced glaucoma, VF damage is often characterized by large arcuate scotomata, which have connected to form a ring, threatening visual function in the central area of the VF [3], [4]. The central VF is especially important because cortical magnification in this area is much larger than in the peripheral region [5], [6] and indeed it has been reported that VF damage in the central area results in disability in various daily tasks [7]. Thus, treatments should be intensified when VF damage threatens the patient's visual function, particularly in the central region.

**Figure 1:**
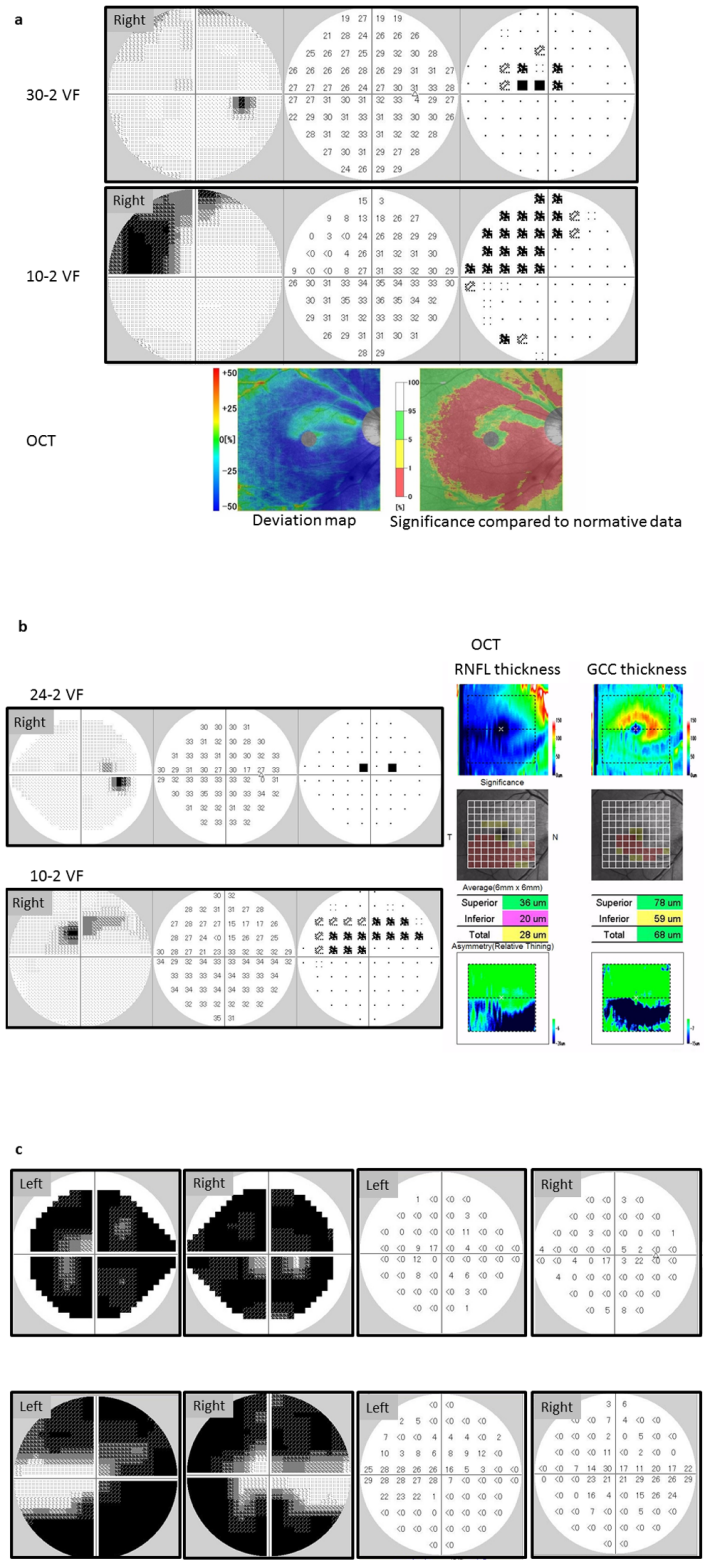
Case examples. Case a: 61-year-old male (right eye, normal tension glaucoma) whose visual acuity was 20/20. The OCT image was obtained using the RS3000 (Nidek Co,.ltd, Gamagori, Aichi, Japan); blue colored regions in the OCT deviation map (left figure) indicate thinner RNFL+GCC than expected; significance is represented in the right figure (significant differences in thickness are colored in red). Case b: 60-year-old male (right eye, normal tension glaucoma) whose visual acuity was 20/20 (right eye). The OCT image was obtained using the 3D OCT-2000 (Topcon Corp, Tokyo, Japan), Case c: 69-year-old male (normal tension glaucoma) whose visual acuities were 20/32 (right eye) and 20/25 (left eye). OCT: Optical coherence tomography. RNFL: retinal nerve fiber layer, GCC: ganglion cell complex.

In glaucoma, VF sensitivities are highly correlated across corresponding regions of the retina [8]–[10] and several maps have been proposed to describe these correlations as clusters in the central 30 degrees [8], [10]–[14]. In most of these maps, test points in the central 10 degrees tend to be clustered in small numbers. However, parafoveal defects can occur preferentially in early glaucoma [15]–[18], probably due to a distinctive pathological mechanism [15], and it has been suggested that patients with paracentral defects cannot be well-monitored unless a central 10° test program is used to densely measure this region [19]–[21]. A recent paper reported that the 24-2 VF is not optimal for detecting early damage of the macula but VF damage in the central area can be detected early in the disease process using the 10-2 VF [22]. Interesting case examples are illustrated in Figure 1; in patients a and b, much deeper VF scotomata are observed in the 10-2 VFs than one might expect from inspection of their 30-2/24-2 VFs; however, the central damage is supported by findings obtained with optical coherence tomography. On the other hand, in case c, a considerable region of the VF can be seen to be preserved in the 10-2 VF, despite the appearance of almost complete blindness in the 24-2 VF; moreover, it is interesting to note that visual acuities in this patient were maintained: 20/32 in the right eye and 20/25 in the left eye. These examples illustrate the motivation for the current study: cluster test points in glaucoma patients' 30-2 and 10-2 VFs in order to map the different regions damaged by the disease.

## Method

This study was approved by the Research Ethics Committee of the Graduate School of Medicine and Faculty of Medicine at the University of Tokyo. Written consent was given by patients for their information to be stored in the hospital database and used for research. This study was performed according to the tenets of the Declaration of Helsinki.

This was a retrospective study that included 128 eyes from 128 patients with a diagnosis of: primary open-angle glaucoma (125 eyes), and secondary open angle glaucoma (pseudoexfoliation; 3 eyes). Patients were followed in the general glaucoma clinic at the University of Tokyo Hospital. Patients who underwent measurements with both the 30-2 and 10-2 VF test patterns (Humphrey Field Analyzer: HFA, Carl Zeiss Meditec, Dublin, CA) in a single day were included. One eye was chosen randomly when both eyes met the criteria; right eye VFs were mirror-imaged to a left eye format. Other criteria for inclusion in the study were visual acuity better than 6/12, no previous ocular surgery (except for cataract extraction and intraocular lens implantation), and no other posterior segment eye disease. All VFs were recorded using the SITA standard strategy with a Goldmann size III target. Reliability criteria applied were fixation losses less than 25% and false-positive responses less than 15%, a false-negative rate was not used to exclude VFs based on results in Bengtsson and Heijl [23].

### Comparison between 30-2 VF and 10-2 VF test results

Mean deviation (MD) values derived from patients' 30-2 VFs and 10-2 VFs were compared using Pearson's correlation coefficient. In addition, Pearson's correlation coefficient was calculated (i) between the mean of the total deviation (TD) values of the four innermost test points in the 30-2 VF (locations [3,3], [3,−3], [−3,3] and [−3,−3]; [x-axis coordinate, y-axis coordinate]) denoted 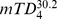 and the mean of the TD values of all 68 test points in the 10-2 VF (denoted 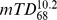), and (ii) between the each of the TD values of the four innermost test points in the 30-2 VF (denoted 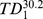) and the mean of the TD values of the innermost 17 test points of the 10-2 VF (denoted 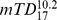) in each of superior-temporal, superior-nasal, inferior-temporal and inferior-nasal quadrants.

### VF clustering analysis

Test points from the 30-2 and 10-2 VFs were clustered using the Hierarchical Ordered Partitioning and Collapsing Hybrid (HOPACH) – Partitioning Around Medoids (PAM) algorithm, for its ability to hierarchically order and partition clusters into finite groups in an unbiased manner. The HOPACH-PAM algorithm is a hybrid between hierarchical ordered partitioning and collapsing [24]–[26]; in other words, HOPACH builds a hierarchical tree of clusters by recursively partitioning the VF, while ordering and possibly collapsing clusters at each level to identify finite structures in a dataset. The HOPACH-PAM algorithm uses the Mean/Median Split Silhouette (MSS) criteria which is particularly apt at identifying structures in a dataset [27]. One of strengths of this approach is that the optimum number of VF clusters is mathematically inferred by the algorithm; this is in contrast to many other clustering approaches, such as k-means and hierarchical clustering methods, that arbitrarily decide the number of optimum clusters, which can lead to incorrect results [24].

Using the HOPACH-PAM algorithm, the 74 test points from the 30-2 VF and 68 points from the 10-2 VF were clustered according to their TD values; the four test points (locations [3,3], [3,−3], [−3,3] and [−3,−3]; [x-axis coordinate, y-axis coordinate]) overlapping between the two test patterns were taken from the 30-2 VF (please note there are no test points at the locations of locations [3,9], [9,3], [−3,9], [−9,3], [3,−9], [9,−3] [−3,−3] and [−3,−9]; [x-axis coordinate, y-axis coordinate] in 10-2 VF). Clustering methods are frequently carried out in an exploratory manner and often the patterns found are not translatable to other datasets [28] hence bootstrap analysis (10,000 re-samples) was performed to explore the significance of the clustering results.

All statistical analyses were carried out using the statistical programming language R (ver. 2.15.1, The R Foundation for Statistical Computing, Vienna, Austria). The R package “hopach” was used to carry out the analysis of HOPACH-PAM.

## Results

Table 1 shows the subjects' demographics.

**Table 1:** Subject demographics.

age (mean ± sd, [range])	58.1±13.2 [21 to 86]
male/female	63/65
Type of glaucoma	
NTG	74
POAG	51
PEG	3
MD (30-2 VF, mean ± sd, [range])	−14.8±7.5 [0.5 to −29.2]
MD (10-2 VF, mean ± sd, [range])	−15.5±7.7 [−0.3 to −30.9]

sd: standard deviation, NTG: normal tension glaucoma, POAG: primary open angle glaucoma, PEG: pseudoexfoliation glaucoma, MD: mean deviation.

**Figure 2:**
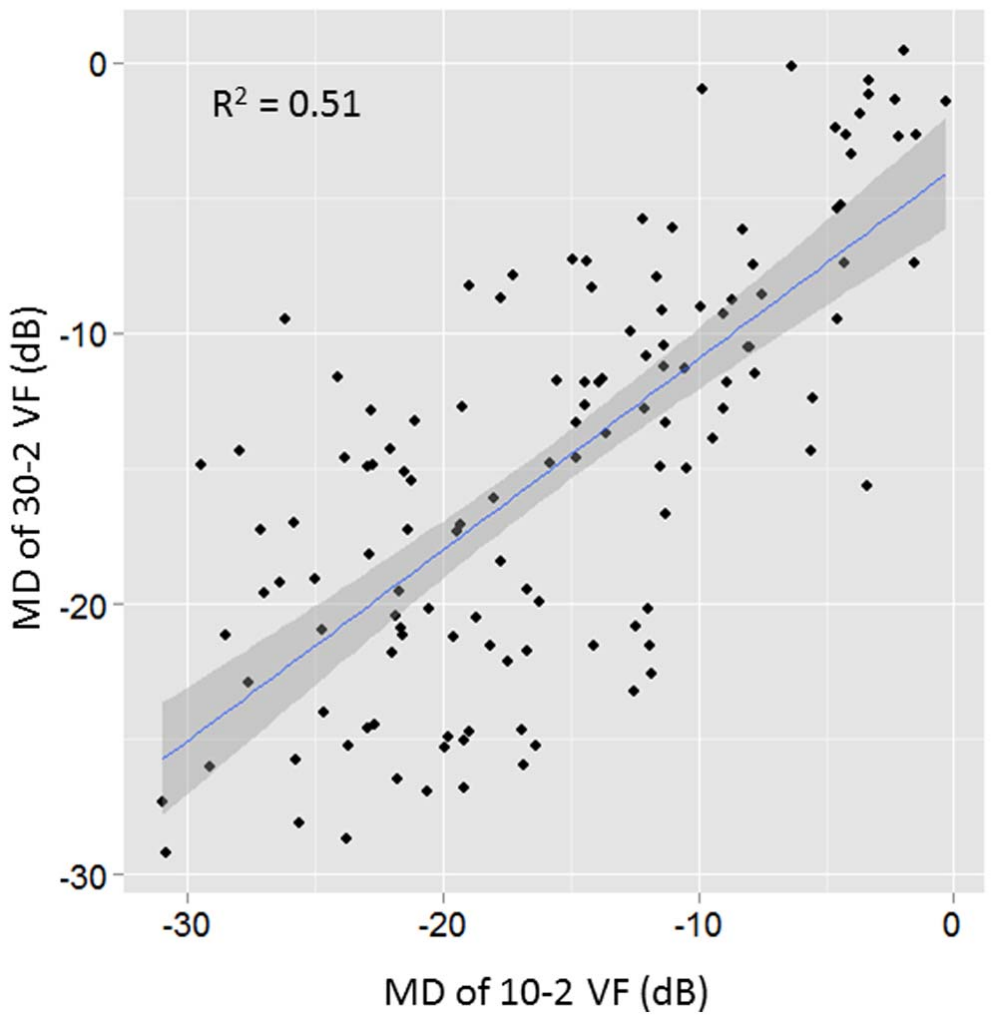
The relationship between the mean TD value of the four most central test points in the 30-2 VF and the mean TD value of all 68 points in the 10-2 VF. There was a significant and strong relationship (y = −3.5+0.84 x, R^2^ = 0.80, p<0.0001). 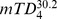: mean of the TD values of all 74 test points in the 30-2 VF and 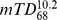: mean of the TD values of all 68 test points in the 10-2 VF. Shaded area corresponds to the 95% confidence interval of the pooled regression.

**Figure 3:**
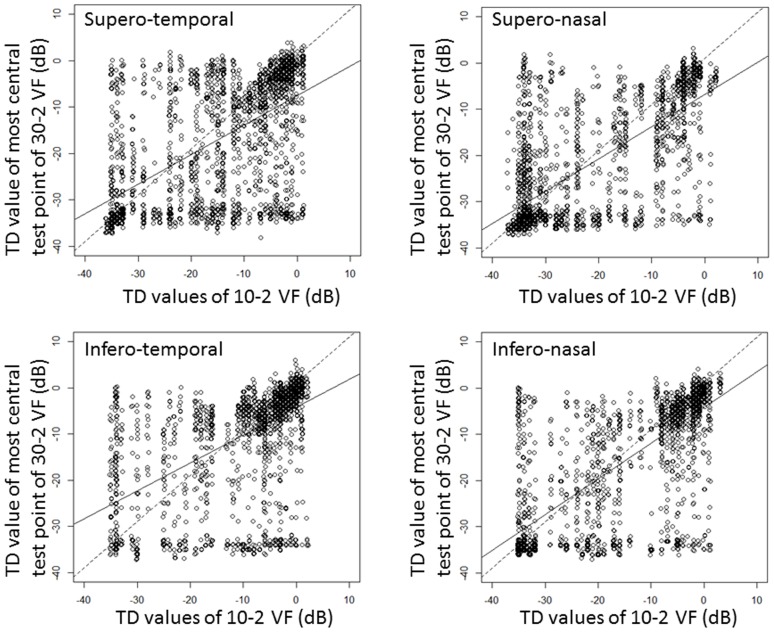
The relationship between the 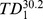
 **and** 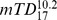 **values in the same quadrant.** Top left: Supero-temporal quadrant (y = −7.5+0.64 x, R^2^ = 0.34, p<0.0001), Top right: Supero-nasal quadrant (y = −6.8+0.70 x, R^2^ = 0.51, p<0.0001), Bottom left: Infero-temporal quadrant (y = −4.2+0.60 x, R^2^ = 0.32, p<0.0001), Bottom right: Infero-nasal quadrant (y = −4.3+0.77 x, R^2^ = 0.56, p<0.0001). 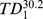: innermost test points in the 30-2 VF, 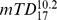: innermost 17 test points of the 10-2 VF.

There was a significant and moderate association between MD values derived from patients' 30-2 VFs and 10-2 VFs (R^2^ = 0.51, p<0.0001). There was also a significant and strong relationship between 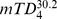 and 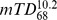 values (R^2^ = 0.80, p<0.0001, Figure 2). Finally, there was a significant but relatively weak correlation between 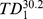 and 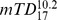 values (R^2^ = 0.34, 0.51, 0.32 and 0.56 (p<0.001) in the superior-temporal, superior-nasal, inferior-temporal and inferior-nasal quadrant, respectively): see Figure 3.

**Figure 4:**
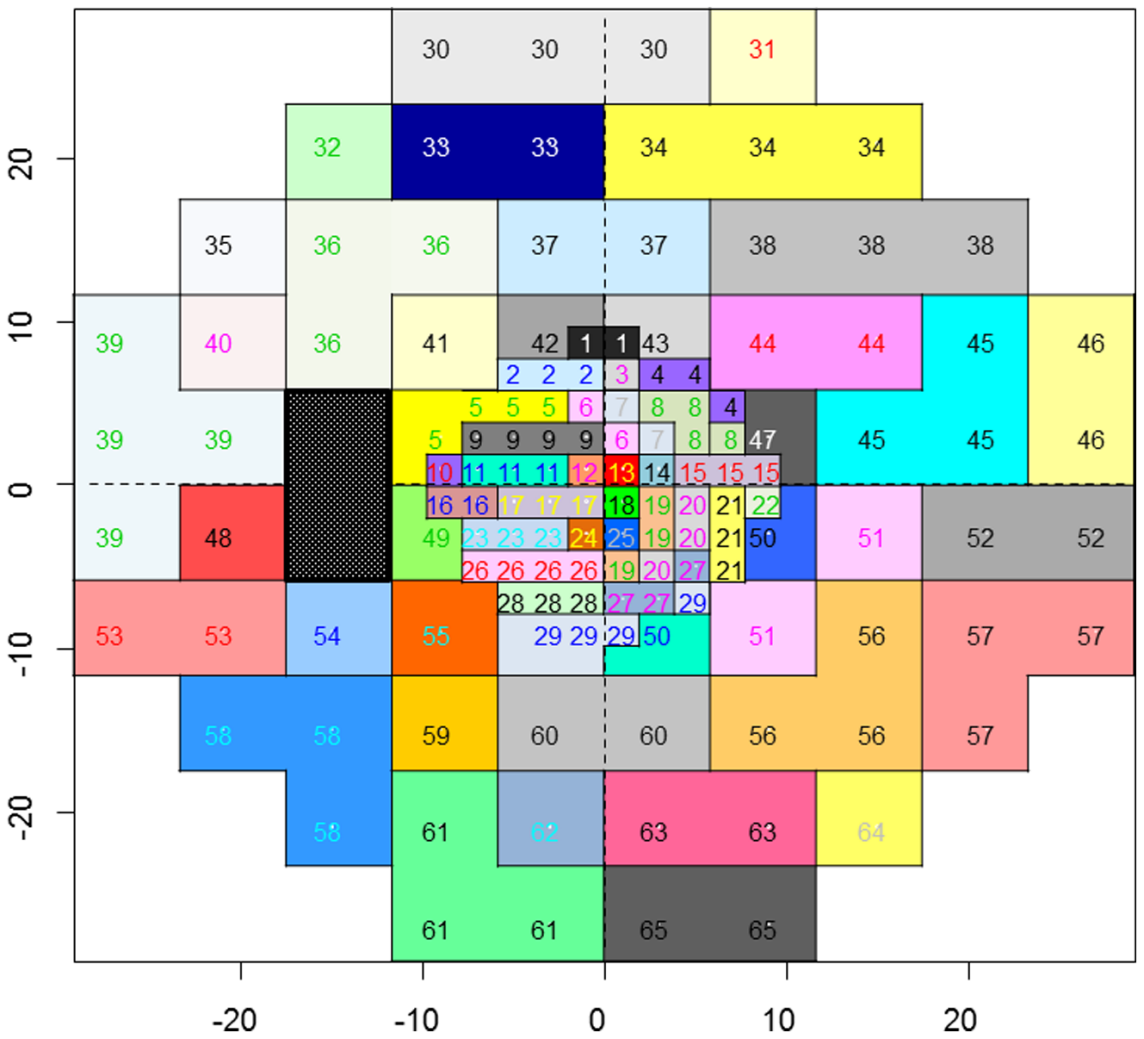
65 sectors obtained from the 30-2 and 10-2 VFs (left eye). There were 38 sectors in the 30-2 VF and 29 sectors in the 10-2 VF. Two test points in the 30-2 VF belong to 10-2 VF sectors (sector 5 and 29). TD values at (X coordinate, Y coordinate) = (3, 3), (3, −3), (−3, 3) and (−3, −3) derived from the 30-2 VF.

**Figure 5:**
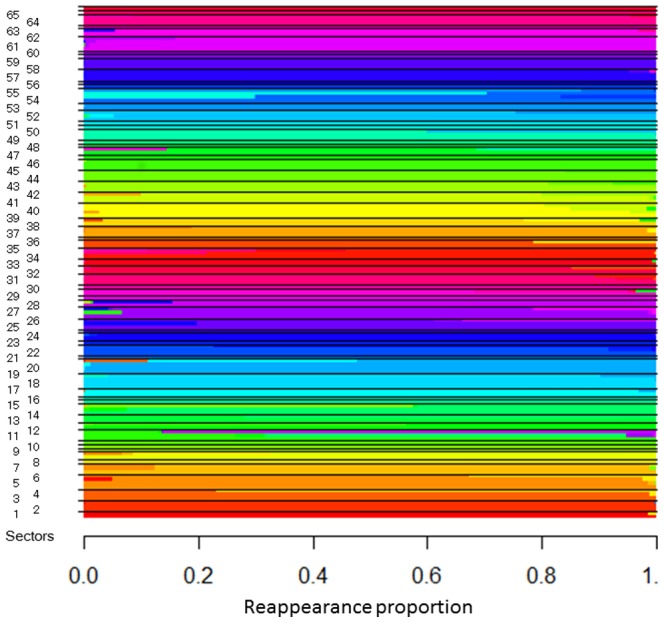
Cluster probability plot. Test points were ordered within a cluster by their reappearance probabilities, obtained by bootstrapping (10,000 times). We can see which clusters are most stable (wide bars) and which pairs of clusters are most likely to exchange test points with one another. Most of the clusters had good stability and the cluster stability did not differ discernibly between 30-2 VF and 10-2 VF sectors.

Results of the HOPACH-PAM clustering are illustrated in Figure 4; 65 clusters (‘sectors’) were identified, adjacent VF test points tended to be clustered into the same sector even when test points were derived from different VF tests (30-2 or 10-2 VF grids). Furthermore, clustering appears to follow the average distribution of the retinal nerve fiber layer (RNFL). Figure 5 illustrates the results of bootstrapping; we can see which clusters are most stable (wide bars) and which pairs of clusters are most likely to exchange test points with one another. Most sectors had good stability and this did not appear to be affected by the particular test pattern.

## Discussion

In this study the relationship between test locations in the 30-2 VF and 10-2 VF was investigated. Although TD values in the 10-2 VF were highly correlated with the central four TD values of the 30-2 VF on average, there are still some instances of large agreement in individual patients. In particular in early glaucomatous cases where 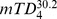 tends to better preserved than 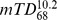 (see Figure 3). In addition, the relationship between 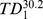 and 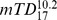 is relatively weak, which indicates that the magnitude of association varies significantly from patient-to-patient. Thus, it appears that the 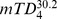 and 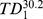 are insufficient to precisely estimate visual defects in this region of the central VF. Among the 65 sectors identified from the HOPACH-PAM clustering algorithm, 38 sectors were located outside the 10-2 VF whereas 29 sectors fell inside the 10-2 VF. Bootstrapping suggested that clusters from both the 10-2 VF and 30-2 VF were stable. Moreover, these sectors appeared to follow the distribution of retinal nerve fiber bundles.

As shown in the Figure 4, a large number of sectors were identified in the 10-2 VF that were completely independent of points in the 30-2 VF. This clearly suggests that clinicians cannot solely rely on the 30-2 VF when evaluating damage in glaucoma patients; closer examination of the central ten degrees, using a denser test grid than currently employed in the 30-2 VF, is essential. Park et al. have suggested that eyes presenting with parafoveal scotomata have distinctly different risk factors to patients with other patterns of VF damage [15]; moreover, Park et al. argue that progression of this defect can be detected more effectively with the 10-2 VF test grid compared with the 30-2 VF [21]. On the other hand, clinicians cannot rely exclusively on the 10-2 VF because typical glaucomatous VF damage such as arcuate scotoma and the nasal step defect [29] would not be detected with this test grid. Hence, it is not recommended to carry out 10-2 VFs as an alternative to 30-2 VF testing but neither is it recommended to alternate 30-2 and 10-2 VF tests, because the detection of progression will, in general, be delayed with a reduced number of consistent test grids. Therefore, clinicians are advised to continue to use the same strategy (e.g., always 30–2 or 24-2 VFs) [30]. Furthermore, VF testing at some hospitals is performed much less than recommended [31], hence alternating test patterns will only compound this problem. Consequently, it may be beneficial to develop a new VF test grid that spans the central 30 degrees but includes a higher density of test points in the central ten degrees; the clustering results presented here could help to devise this new test pattern.

A possible caveat of the current study is the sample of patients studied; in particular, patients may have been undergone 10-2 *and* 30-2 VF tests on the same day because the clinician suspected a paracentral defect that had not been detected using the 30-2 VF test. Nonetheless, the purpose of our study was to investigate whether the spatial sampling of the 30-2 VF is sufficient to detect paracentral damage, which is greatly important for VRQoL.

Several studies have clustered 30-2 or 24-2 VFs in order to inform the relationship between test points [8], [12], [13], the anatomical structure of retinal nerve fiber bundles [14] and the pattern of progression rates [11]; these studies, like the one presented here, have all identified clusters that to some extent follow the structural distribution of RNFLs. In addition, Koseki et al. have clustered points in the 10-2 VF [32], revealing groups of points that also appear to follow the paths of RNFLs. Our results are in agreement with these studies, with clusters identified in both the 10-2 VF and the 30-2 VF that follow the distribution of the RNFL. Noteworthy in the current results is the large number of 10-2 VF clusters relative to the number of clusters from the 30-2 VF; in addition, the stability of 10-2 VF clusters (as revealed by bootstrapping) was, in general, as stable as clusters in the 30-2 VF. These findings suggest that many 10-2 VF clusters exist independently from clusters in the 30-2 VF. In our results, no clusters spanned the meridian line; this outcome is in good agreement with the anatomical distribution of the RNFL [33]. One exception, however, is sector 39, which is located in the temporal area of the VF; we suppose that the sparse distribution of RNFL in this area and low frequency of glaucomatous VF deterioration in this region is one explanation for this finding. It would be interesting to further investigate the separation of the anatomical upper and lower hemifields in the temporal VF area.

Glaucomatous RNFL damage predominantly occurs in the supero- and infero-temporal angles around the optic nerve head. Hood et al. [33] and Heijl et al. [16] have both reported that glaucomatous VF damage usually commences in the central VF, particularly in the superior hemifield; Hood et al. have suggested that this is because most of the corresponding RNFLs flow into the optic disc at the infero-temporal angle. Supporting this suggestion is the observation that early glaucomatous VF change occurs in this area almost as frequently as the ‘classic’ arcuate defect and nasal step defect [18], [34], [35]. Furthermore, the density of RNFLs is very high in this region and despite the fact that it represents less than 2% of the retinal area it contains more than 30% of ganglion cell [36]. Consequently, it is perhaps not surprising to see so many clusters in the central superior hemifield. On the other hand, the central inferior VF area tends to be preserved until late stage glaucoma. Hood et al. have suggested this is because the RNFLs in this area penetrate the optic disc margin at the temporal angle, which is usually less likely to be affected in early to moderate glaucoma [19]. As a consequence, the clusters identified in the inferior central hemifield may be a result of VF damage observed in a subsample of late stage glaucoma patients. Interestingly, Hood et al. have suggested superior VF test points, just above the horizontal line, tend not to be involved in early to moderate glaucomatous VF change; accordingly, Sectors 10, 11 and 12 are probably a consequence of VF change in advanced glaucoma patients, similarly to clusters in the central inferior VF area. As shown in Figure 5, most of the identified sectors were reproduced in the same area in the bootstrapped samples. However, some sectors, such as 11, 12, 21, 28, 37, 54 and 55, were not stable. As structure-function mapping is influenced by many ocular parameters, such as position of the ONH in relation to the fovea, disc area, axial length, spherical equivalent, disc shape, disc orientation and disc tilt [37], it is not surprising that no one clustering result will be applicable to all patients. Future studies should be carried out to continue efforts to create patient-customized VF cluster maps that consider these parameters.

In conclusion, this study suggests that many areas in the 10-2 VF as well as the 30-2 VF are affected by glaucoma. Thus, it is not sufficient to merely measure a glaucoma patient's 30-2 VF; instead it should be recommended to additionally measure a dense test grid, such as the 10-2 VF pattern, when evaluating the status of VF damage.

## Supporting Information

Data S1 **Visual field data analyzed.**
(CSV)
